# Reversed Neurovascular Coupling on Optical Coherence Tomography Angiography Is the Earliest Detectable Abnormality before Clinical Diabetic Retinopathy

**DOI:** 10.3390/jcm9113523

**Published:** 2020-10-31

**Authors:** Yi Stephanie Zhang, Ilda Mucollari, Changyow C. Kwan, Gianna Dingillo, Jaspreet Amar, Gregory W. Schwartz, Amani A. Fawzi

**Affiliations:** 1 Department of Ophthalmology, Feinberg School of Medicine, Northwestern University, Chicago, IL 60611, USA; yistephaniezhang@gmail.com (Y.S.Z.); ildamucollari2022@u.northwestern.edu (I.M.); clairekwan29@gmail.com (C.C.K.); gmd52@case.edu (G.D.); jaspreet.amar@my.rfums.org (J.A.); greg.schwartz@northwestern.edu (G.W.S.); 2 Department of Physiology, Feinberg School of Medicine, Northwestern University, Chicago, IL 60611, USA

**Keywords:** neurovascular coupling, OCT angiography, diabetic retinopathy, dark adaptation

## Abstract

Diabetic retinopathy (DR) has traditionally been viewed as either a microvasculopathy or a neuropathy, though neurovascular coupling deficits have also been reported and could potentially be the earliest derangement in DR. To better understand neurovascular coupling in the diabetic retina, we investigated retinal hemodynamics by optical coherence tomography angiography (OCTA) in individuals with diabetes mellitus (DM) but without DR (DM no DR) and mild non-proliferative DR (mild NPDR) compared to healthy eyes. Using an experimental design to monitor the capillary responses during transition from dark adaptation to light, we examined 19 healthy, 14 DM no DR and 11 mild NPDR individuals. We found that the only structural vascular abnormality in the DM no DR group was increased superficial capillary plexus (SCP) vessel density (VD) compared to healthy eyes, while mild NPDR eyes showed significant vessel loss in the SCP at baseline. There was no significant difference in inner retinal thickness between the groups. During dark adaptation, the deep capillary plexus (DCP) VD was lower in mild NPDR individuals compared to the other two groups, which may leave the photoreceptors more susceptible to ischemia in the dark. When transitioning from dark to ambient light, both diabetic groups showed a qualitative reversal of VD trends in the SCP and middle capillary plexus (MCP), with significantly decreased SCP at 5 min and increased MCP VD at 50 s compared to healthy eyes, which may impede metabolic supply to the inner retina during light adaptation. Mild NPDR eyes also demonstrated DCP dilation at 50 s and 5 min and decreased adjusted flow index at 5 min in light. Our results show altered neurovascular responses in all three macular vascular plexuses in diabetic subjects in the absence of structural neuronal changes on high resolution imaging, suggesting that neurovascular uncoupling may be a key mechanism in the early pathogenesis of DR, well before the clinical appearance of vascular or neuronal loss.

## 1. Introduction

Diabetic retinopathy (DR) is the leading cause of vision impairment and blindness in the world and in working-aged adults in the United States [1,2]. Understanding the pathogenesis of DR has been a challenging yet critical area of investigation. Clinically, DR is characterized by microvascular abnormalities, progressing from microaneurysms in mild non-proliferative DR (mild NPDR) to intraretinal hemorrhages, exudates, macular edema, and eventually aberrant preretinal neovascularization [3]. Additional support for a primary vascular pathogenesis include evidence from optical coherence tomography angiography (OCTA) studies that show capillary loss and foveal avascular zone (FAZ) enlargement that may be detectable before the appearance of clinical retinopathy and progress relentlessly with advancing retinopathy severity [4,5,6,7,8,9,10]. On the other hand, the opposing view posits that retinal neuropathy is the primary pathogenic driver of DR, as evidenced by structural inner retinal thinning and ganglion loss [11,12] as well as neural dysfunction on electroretinogram [13,14] in the diabetic retina prior to detectable vascular alterations [15,16]. However, these primary vascular or neuronal alterations are not universal in diabetic eyes without retinopathy (DM no DR), with many studies reporting normal vasculature on OCTA, unchanged inner retinal thickness, and no dysfunction on ERG [9,17,18].

Though much of the current debate has centered on whether DR is a primary microvasculopathy or neuropathy, disruption of neurovascular coupling has also been reported in diabetes. However, until recently, these responses have been inaccessible to detailed study in three-dimensional retinal capillaries [19,20,21,22]. Neurovascular coupling is the physiologic autoregulation of blood flow in the brain and the retina in response to local neuronal metabolic demand [23,24,25,26]. Within the neurovascular unit, neuronal and glial cell signals elicit vascular responses mediated by contractile pericytes and responsive endothelial cells in order to maintain appropriate perfusion [26]. This process is thought to be orchestrated by the complex interaction between neurons, glia and microvascular cells, guided by feedback mechanisms from neuronal metabolites and feedforward signals through neurotransmitters and vasoactive agents [25,26,27]. However, this process is known to be disturbed in the brain as well as in the retina of diabetic individuals [19,20,21,28,29].

Diabetic retinal pathology affects the entire neurovascular unit, with evidence of loss of inner retinal neurons and pericytes, basement membrane thickening, as well as dysregulation of nitric oxide, a mediator of neurovascular coupling [2,3,26,30,31,32]. Grunwald et al. had previously shown an attenuation of retinal vasoconstriction, a response found in healthy controls, to hyperoxia that worsened with increasing severity of DR [29]. In addition, in response to flicker stimulation, which increases neuronal metabolic demand, the diabetic retina has a significantly curtailed arteriolar vasodilation as shown on retinal vessel analyzer and laser Doppler flowmetry [21,22,33,34].

More recently, OCTA investigations of the three macular capillaries has revealed distinct neurovascular coupling responses in each of these vascular plexuses in the retina. These dynamic responses were monitored while altering the subject’s light adaptation and with it, the neuronal metabolic demand of individual retinal layers [2,35,36,37,38]. Using OCTA in this fashion has allowed us to gain a more detailed view of the three-dimensional macular capillary responses than ever before. In fact, with this approach, our group has recently shown that healthy individuals challenged with oral glucose tolerance solution demonstrate complete reversal of the macular capillary responses to modulated light conditions [37].

These studies stimulated us to wonder whether neurovascular coupling deficits could be the earliest pathological indicators in DR, before static microvascular or structural neuronal changes are visualized. To address our question, we used OCTA to examine the dynamic course of neurovascular coupling, as well as the structural capillary vessel density and neural ganglion cell complex thickness in eyes with DM no DR and mild NPDR, and compared them to healthy controls.

## 2. Materials and Methods

This prospective study took place from June to October 2019 in the Department of Ophthalmology at Northwestern University in Chicago, Illinois. Study approval (STU00200890) was obtained from the Institutional Review Board of Northwestern University and conducted in accordance with the tenets of the Declaration of Helsinki and the Health Insurance Portability and Accountability Act regulations. Written informed consent was obtained from all participants.

### 2.1. Study Sample

We recruited a total of 20 control, 15 DM no DR, and 13 mild NPDR participants from the ophthalmology clinic at Northwestern Memorial Hospital. Inclusion criteria included either healthy individuals or treatment-naïve patients with DM no DR or mild NPDR. These diagnoses were initially based on chart review of dilated fundus examination findings within the preceding 3 months. Subsequently, these diagnoses were confirmed in each subject by fundus photographs using an ultra-widefield scanning laser ophthalmoscope (Optomap Panoramic 200; Optos PLC, Scotland, UK) within 2 weeks of participation in OCTA imaging experiments. Two separate graders (Y.S.Z. and C.C.K) and a retina specialist (A.A.F.) confirmed the final grading of DR stage on color fundus photographs based on the International Clinical Diabetic Retinopathy Disease Severity Scale [39]. On the day of OCTA imaging, additional inclusion criteria included blood pressure ≤ 140/90 mmHg and blood glucose ≤ 160 mg/dL. In our image analysis, we only included images with a signal strength index (SSI) of ≥50, quality index (Q-score) of ≥6, decentration ≤5% of the parafoveal area, and no significant motion or shadow artifacts.

Participant past medical history was obtained by subject report and review of electronic heath records. We excluded individuals with a history of significant vascular disease such as uncontrolled hypertension, stroke, or significant smoking. Patients with a history of prior retinal treatments such as intravitreal injections, focal laser and/or panretinal photocoagulation, or pars plana vitrectomy were excluded. Other exclusion criteria included a history of ocular disease such as glaucoma, media or lens opacities, or refractive error greater than 6.0 diopters.

### 2.2. Image Acquisition

We obtained 3 × 3 mm^2^ OCTA angiograms and structural OCT images centered on the fovea using the RTVue-XR Avanti system with split-spectrum amplitude-decorrelation angiography (SSADA) algorithm and 3D projection artifact removal (3D-PAR) technology (Optovue Inc., Fremont, CA, USA. Software Version 2017.1.0.151) [40]. Briefly, the system used a light source centered on 840 nm to capture 70,000 A-scans per second and two consecutive B-scans, each containing 304 A-scans at a sampling location on the retina. The SSADA algorithm then obtained angiographic flow information from the OCTA reflectance decorrelation between the two consecutive B-scans.

The machine provided the signal strength index (SSI), which is a value of the scan’s reflectance signal strength, and a quality index (Q-score) which reflects the overall image quality based on a variety of factors including SSI, focus, and motion artifacts [41,42].

### 2.3. Dark to Light Adaptation Imaging Protocol

OCTA imaging was performed on one eye of each subject using our previously reported dark to light adaptation protocol [35,36,37]. Before the experiment, participants were asked to fast for 10 h overnight. Insulin-dependent participants were instructed to continue their usual home regimen including basal insulin the night before the experiment. No insulin was administered the morning of the experiment. All experiments were completed prior to noon to minimize diurnal variation. Prior to commencing the imaging session, blood pressure and blood glucose were measured. Blood pressure measurements were converted from systolic and diastolic values to mean arterial pressure (MAP) [43].

**Figure 1:**
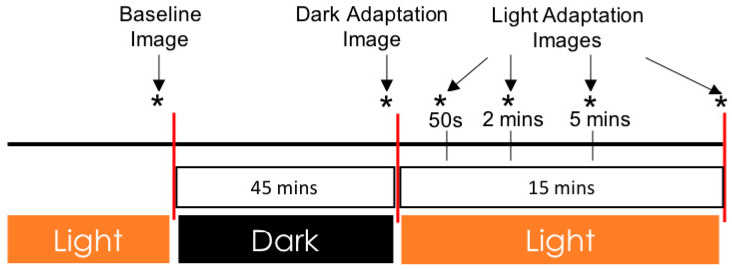
Schematic of dark and light adaptation image acquisition. Images were acquired at baseline in ambient light, after 45 min of dark adaptation, and at several time points (50 s, 2 min, 5 min, and 15 min) after transitioning from dark to ambient light. An asterisk (*) denotes a timepoint of optical coherence tomography angiography (OCTA) image acquisition.

The imaging protocol is summarized in Figure 1. At the beginning of the experiment, prior to any adaptation, participants underwent baseline OCTA imaging in ambient light. Subsequently, in the dark adaptation protocol, participants wore a thick eye patch over the right eye in a completely dark room for 45 min. Participants who had fixation difficulties with the right eye, or whose left eye had a more advanced retinopathy grade, underwent the imaging protocol of the left eye. At the end of the dark adaptation, the OCTA computer monitor was turned on and adjusted to display only red light. The participants subsequently removed their eye patch and underwent dark-adapted OCTA image acquisition. Following the dark-adapted scan, the room light was turned on to ambient levels (800 cd/m^2^) and a scan was acquired at four consecutive time points in the light (50 s, 2 min, 5 min, and 15 min).

### 2.4. Image Analysis

**Figure 2:**
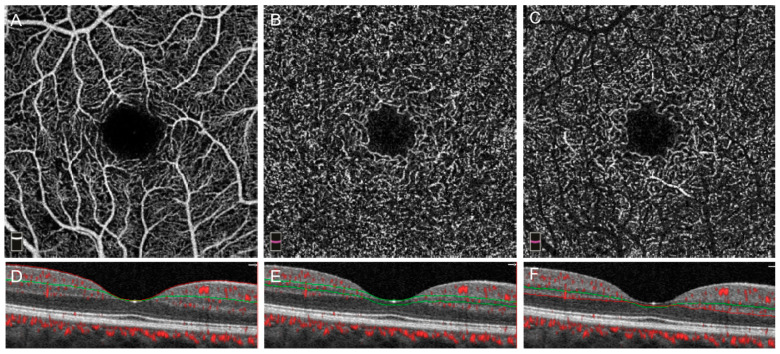
OCTA image segmentation. Panels (**A**–**C**) show the respective en-face angiograms of the superficial, middle, and deep capillary plexuses with cross-sections shown in (**D**–**F**) with angio overlay.

We manually segmented the OCTA angiograms into the superficial capillary plexus (SCP), middle capillary plexus (MCP), and deep capillary plexus (DCP) as shown in Figure 2 [10,44]. SCP and full retinal angiograms were obtained from the built-in AngioVue Analytics software (Optovue Inc., Fremont, CA, USA. Software Version 2017.1.0.151). The machine automatically segmented the SCP from the internal limiting membrane (ILM) to 10 µm above the inner plexiform layer (IPL), and the full retinal thickness angiograms from the ILM to 10 μm below the outer plexiform layer (OPL). The full retina angiograms were used to define the signal-to-noise threshold value in subsequent manual image analysis. We manually segmented the MCP and DCP using boundaries of 10 μm above to 30 μm below the IPL and 30 µm below the IPL to 10 µm below the OPL, respectively [44].

**Figure 3:**
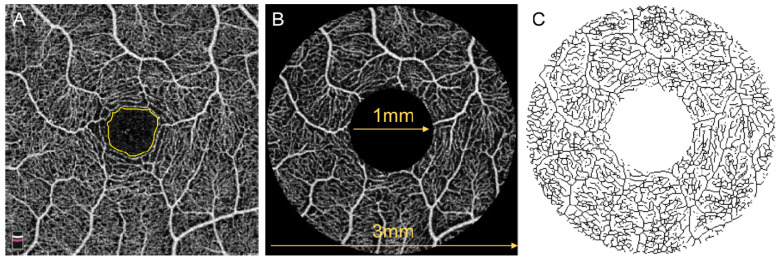
Schematic of OCTA parameters. (**A**) Full retinal thickness OCTA image of a healthy control showing the foveal avascular zone (FAZ) outlined in yellow. The FAZ was used to establish the background threshold for both vessel length density (VLD) and adjusted flow index. (**B**) Superficial capillary plexus (SCP) angiogram of the same individual showing delineation of the parafovea area between two rings of 1 mm and 3 mm in diameter. All OCTA parameters were obtained for the parafovea. (**C**) Skeletonized SCP image for the calculation of vessel length density (VLD) after binarization based on a threshold determined by the FAZ and subsequent skeletonization of all vessels to 1-pixel wide.

OCTA parameters were obtained for the parafoveal region, defined as the annular area centered on the fovea enclosed by inner and outer ring diameters of 1 mm and 3 mm, respectively (Figure 3B). The built-in software calculated the vessel density (VD) for the three vascular plexuses based on the percentage of pixels in the parafovea occupied by vessels. Parafoveal vessel length density (VLD) and adjusted flow index (AFI) measurements were calculated on ImageJ [45] by two separate graders (Y.S.Z., I.M.) using previously described methods [4,46]. The images were binarized based on the FAZ of the full retinal thickness angiograms (Figure 3A) in order to differentiate true vessel signal from background noise.

VLD is obtained by skeletonizing all vessels to 1-pixel wide on ImageJ and is defined as the total length occupied by superficial vessels in the parafovea (Figure 3C). We calculated the SCP VLD as the percentage of pixels covered by skeletonized vessels in the parafovea. Because all vessels are 1-pixel wide, the VLD removes the disproportionate influence of larger superficial vessels in the VD measurement. AFI is an indirect measure of flow velocity based on the average decorrelation value of the angiogram [47]. We calculated AFI in the three vascular plexuses on ImageJ as the average pixel intensity of parafoveal vessels above the background threshold based on the FAZ noise level.

In addition to angiographic information, we also obtained structural inner retinal ganglion cell complex (GCC) thickness data from the OCTA machine. Parafoveal GCC thickness is measured by the machine from the ILM to below the IPL.

### 2.5. Statistical Analysis

Since we are interested in changes in vascular response to new illumination conditions, in our data analysis we converted the OCTA parameters obtained in the dark from their absolute numeric values into relative values to the baseline, with the baseline set as zero. Our OCTA parameters in ambient light were also converted from absolute to their relative values in relation to the dark-adapted state, with the measurements taken in the dark set to zero. For example, relative SCP AFI at 2 min in ambient light would be calculated as SCP AFI_2 min_–SCP AFI_dark_.

All statistical analysis was performed on SPSS version 26 (IBM SPSS Statistics; IBM Corporation, Chicago, IL, USA). Intergrader reliability for AFI and VLD measurements were evaluated by two-way random intraclass correlation coefficient (ICC) analysis. Shapiro-Wilks test was used to determine normality. Analysis of variance (ANOVA) for parametric and Kruskal-Wallis for non-parametric data were used to compare the age, Hemoglobin A1c (HbA1c), blood glucose, disease duration, and MAP of the three groups of subjects. Pearson and Spearman correlations were used to assess the relationship between OCTA parameters in the dark or in ambient light and potential confounding variables including age, MAP, and Q-score.

To account for potential confounding factors, we performed analysis of covariance (ANCOVA) while adjusting for age and Q-score and applied Benjamini-Hochberg post-hoc corrections for multiple comparisons. We used ANCOVA to compare GCC thickness as well as the absolute and percent change in OCTA parameters (VD, VLD, AFI) at various illumination conditions between the three subject groups. A *p* value of <0.05 was considered statistically significant.

## 3. Results

We initially recruited 20 healthy, 15 DM no DR, and 13 mild NPDR participants for imaging. After imaging, we excluded one healthy, one DM no DR and two mild NPDR participants for poor image quality due to excessive motion artifacts at multiple imaging timepoints. Ultimately, 19 healthy, 14 DM no DR, and 11 mild NPDR participants had sufficient image quality to be evaluated in the study. Within the data from eligible participants, images with poor quality at a single time point were excluded only in that time point with no participant having more than a single image excluded. Of the healthy eyes, three total images (from different individuals) were excluded at different timepoints due to motion artifacts. Another healthy image was excluded due to decentration. Of the DM no DR images, three total images (from different individuals) were excluded for poor image quality, fixation issues, or significant shadow artifacts. Of the mild NPDR images, two images were excluded for poor image quality or missing data.

Demographic information for all participants is summarized in Table 1. The ages of our three groups were statistically similar. MAP was significantly higher in the DM no DR group compared to healthy controls. Comparing the two diabetic groups, the mild NPDR group had significantly higher HbA1C (by laboratory report) and fasting glucose (measured on the day of imaging after fasting overnight). Both diabetic groups had significantly higher measured fasting glucose than controls. 

The ICC comparing the measurements completed by the two independent graders was 0.997 for AFI and 0.934 for VLD. Pearson and Spearman correlation revealed a significant correlation between all OCTA parameters and both age and Q-score. No significant correlation was found between MAP and OCTA parameters.

**Figure 4:**
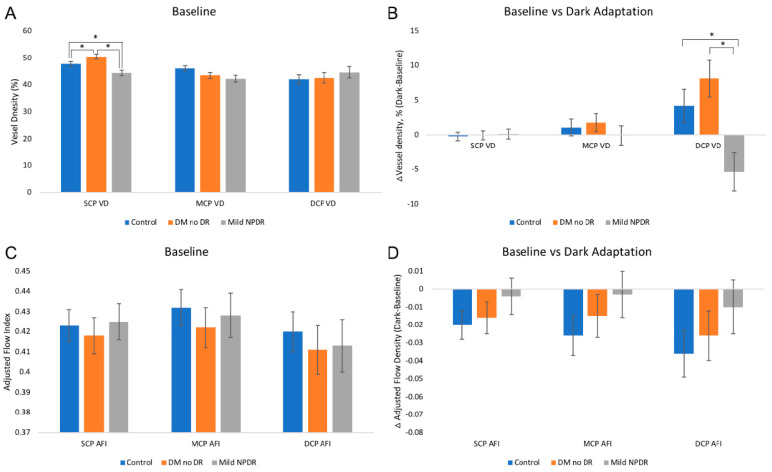
Parafoveal vessel density and adjusted flow index on OCTA at baseline and after dark adaptation. (**A**) Absolute vessel density (VD) and (**C**) adjusted flow index (AFI) at baseline in ambient light before any adaptation. At baseline, the mild NPDR group had significantly decreased superficial capillary plexus (SCP) VD compared to the control and DM no DR groups. The DM no DR group also had significantly higher VD than controls. VD and AFI after dark adaptation in (**B**,**D**) respectively are shown as values relative to baseline, which was set to zero. (**B**) shows significantly lower change in deep vascular plexus (DCP) VD in the mild NPDR group compared to the control and DM no DR groups who had increased DCP VD during dark adaptation. All timepoints with statistically significant differences (*p* < 0.05) between groups are marked with an asterisk (*). Error bars represent standard errors. Abbreviations: VD—vessel density, AFI—adjusted flow index SCP—superficial capillary plexus, MCP—middle capillary plexus, DCP—deep capillary plexus.

**Figure 5:**
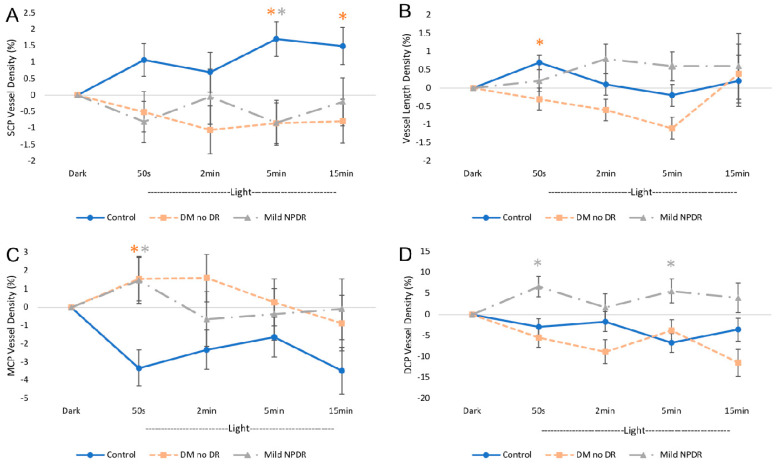
Parafoveal vessel density on OCTA during ambient light transition. Vessel density (VD) in ambient light at each timepoint in (**A**–**D**) are shown as values relative to the dark, which was set to zero. (**A**,**C**,**D**) show VD changes in the superficial (SCP), middle (MCP), and deep (DCP) vascular plexuses, respectively, at consecutive timepoints in ambient light after dark adaptation and comparing the control, DM no DR, and mild NPDR groups. (**A**) SCP VD is significantly increased at 5 min of ambient light in the control compared to the DM no DR and mild NPDR groups, which continues until 15 min in the control compared to the DM no DR group. (**B**) Vessel length density (VLD) measurements indicate significantly elevated VLD in the control compared to the DM no DR group at 50 s in ambient light. (**C**) In the middle capillary plexus (MCP), the two diabetic groups showed significantly increased VD at 50 s in ambient light compared to controls. (**D**) In the deep capillary plexus (DCP), the mild NPDR group showed significantly increased VD at 50 s and 5 min in ambient light compared to controls and to DM no DR. All timepoints with statistically significant differences (*p* < 0.05) between the diabetic and control conditions are marked with an asterisk (*) and the color of the asterisk corresponds with the respective diabetic group that is different from the control. Error bars represent standard errors. Abbreviations: SCP—superficial capillary plexus, MCP—middle capillary plexus, DCP—deep capillary plexus, s—seconds, min—minutes.

**Figure 6:**
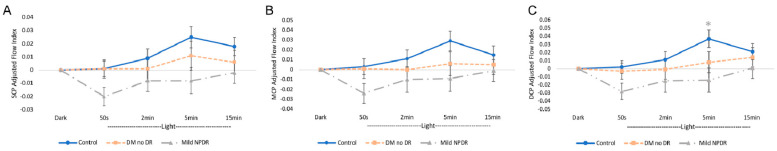
Parafoveal adjusted flow index on OCTA during ambient light transition. Adjusted flow index (AFI) in ambient light at each timepoint in (**A**–**C**) are shown as values relative to the dark, which was set to zero. (**A**) demonstrates ambient light AFI changes in the superficial capillary plexus of the three groups. (**B**,**C**) show a general trend of an increase in AFI in ambient light that peaks earlier and higher (at 5 min) in the middle, and deep capillary plexuses of the control group and later and lower in the diabetic groups. In the deep capillary plexus (DCP), the peak AFI at 5 min in the control group is significantly higher than the DCP AFI of the mild NPDR group. All timepoint(s) with statistically significant differences (*p* < 0.05) between the diabetic and control conditions are marked with an asterisk (*) and the color of the asterisk corresponds with the respective diabetic group that is different from the control. Error bars represent standard errors. Abbreviations: SCP—superficial capillary plexus, MCP—middle capillary plexus, DCP—deep capillary plexus, s—seconds, min—minutes.

Comparisons of the OCT GCC thickness and OCTA parameters between the three groups, while controlling for age and Q-score and adjusting for multiple comparisons, are summarized in Supplementary Tables S1 and S2 and visualized in Figure 4, Figure 5 and Figure 6. There were no differences in GCC thickness between the three groups at baseline (Supplementary Table S1). In contrast, angiographic baseline data showed that mild NPDR eyes had significantly lower SCP VD and VLD than control and DM no DR groups, while DM no DR had significantly higher SCP VD than the control group (Supplementary Table S1, Figure 4A). None of the other capillary layers showed differences at baseline. In the dark, the mild NPDR group demonstrated a significant decrease in DCP VD compared to the control and DM no DR groups that showed increased DCP VD relative to baseline (Figure 4B). There were no statistically significant differences in AFI changes between the three groups in the dark (Figure 4C,D).

With transition to ambient light from dark adaptation, we compared the OCTA parameters between the three groups relative to their respective dark-adapted states (Supplementary Table S2, Figure 5 and Figure 6). In the light, the diabetic groups showed attenuation and reversal of VD and AFI changes in the three vascular plexuses compared to controls. In the controls, qualitative trends in ambient light demonstrated increased AFI in all three plexuses and increased SCP VD, while MCP and DCP VD decreased (Figure 5 and Figure 6). However, in DM no DR eyes, the general trend of VLD, SCP VD, and MCP VD responses to light were reversed in direction and this reversal was statistically significant during at least one timepoint in ambient light (Figure 5). In the SCP, VLD decreased significantly at 50 s, and the VD decreased at 5 and 15 min (Figure 5A,B). In the MCP, VD increased significantly at 50 s in the light compared to controls (Figure 5C). Though not statistically significant, general trends seem to demonstrate delays in the peak of MCP and DCP AFI in ambient light at 15 min in the DM no DR compared to at 5 min in the controls (Figure 6). Mild NPDR patients showed a similar reversal in SCP VD and MCP VD with significantly decreased SCP VD at 5 min and increased MCP VD at 5 min compared to controls (Figure 5A,C). In addition, mild NPDR also exhibited a reversal of DCP responses with significantly increased DCP VD at 50 s and 5 min in the light (Figure 5D). AFI trends showed a general reversal in the mild NPDR group with significantly decreased DCP AFI at 5 min in ambient light compared to control individuals (Figure 6).

## 4. Discussion

In this study, we used OCTA to investigate the hemodynamic responses of the three macular vascular plexuses during dark adaptation and transition to ambient light in patients with DM no DR and mild NPDR compared to healthy individuals. Overall, our results show neurovascular coupling alterations in the three macular vascular plexuses with DCP constriction in the dark in mild NPDR (Figure 4B) and SCP constriction in both diabetic groups during the transition to light, which was significant at 5 min (Figure 5A), and may potentially limit flow across all three vascular layers (Figure 6).

Our baseline data, collected in ambient light before dark adaptation showed significantly lower SCP VD and VLD in the mild NPDR group compared to DM no DR and controls, but no significant differences in other vascular layers. This is in accordance with previous OCTA studies in diabetics showing decreased superficial and deep VD as well as SCP VLD that progressed with increasing severity of DR [4,48,49,50]. However, we did not find changes in DCP OCTA parameters between the groups, which we attribute to our segmentation of the MCP and DCP and controlling for Q-score, which were not addressed by previous researchers [4,48,49,50]. Our results also show an increased SCP VD in the DM no DR group compared to controls. While some previous studies showed decreased VD and VLD in the DM no DR group compared to controls [4,51], Rosen et al. [9] found a significantly increased capillary density and a trend of increased total vessel density in the DM no DR group on OCTA similar to our results. Using adaptive optics scanning laser ophthalmoscopy (AOSLO) to study blood flow in the macula, our group [8] also found higher flow speed and volume in the DM no DR group, with a significant decrease in both metrics in the later stages of DR. In the context of these studies, the increased vessel density in DM no DR individuals could suggest that DM individuals in early disease may exhibit potential compensatory dilation of capillaries, enlistment of reserve capillaries, and increased flow [8,9]. In addition, we propose that the discrepancies with prior findings can be explained by variable DR grading based on clinical versus imaging criteria [52], as well as by the wide spectrum of individuals presenting with DM no DR with a potential compensatory increased VD in early disease and late capillary loss as the disease progresses.

In the dark, participants with mild NPDR demonstrated a decrease in DCP VD from baseline that was significantly lower compared to the control and DM no DR groups, who both showed an increase in DCP VD (Figure 4B). In the dark, photoreceptors experience their highest metabolic demand [53], which is partially met by the DCP of the retinal circulation that supplies the outer retina to complement oxygen diffusion from the choroid [24,54,55]. Previous studies have shown increased central retinal blood velocity on laser Doppler [56,57,58] and increased retinal vessel diameter [57] in the dark, which has been hypothesized to accommodate the heightened oxygen demand of the photoreceptors. Furthermore, our group has shown significant DCP dilation on OCTA in healthy individuals during dark adaptation before transition to ambient light [35,36,37]. Therefore, the finding of decreased DCP VD in diabetic individuals with mild NPDR may suggest disproportionate DCP constriction (or failure to dilate) in response to the oxygen demand in the dark, which may leave the photoreceptors inadequately perfused.

Interestingly, healthy individuals challenged with acute hyperglycemia also exhibit a similarly compromised DCP VD response in the dark compared to the normoglycemic response [37]. It is possible that these healthy individuals also experience relative hypoxia in the DCP during bouts of hyperglycemia. Alternatively we postulate that due to increased choroidal perfusion during hyperglycemia in healthy individuals [59], there is more oxygen in the outer retina, leading to an appropriate DCP constriction. The DCP constriction in healthy subjects challenged with hyperglycemia and in our mild NPDR group suggests that in diabetes, while fasting, the neurovascular response is disrupted in a similar way to a state of acute hyperglycemia in healthy subjects. We cannot rule out the possibility that the diabetic choroid is chronically hyper-perfused due to persistently elevated blood glucose levels, which may explain the DCP responses that we see, though several studies have reported decreased choroidal perfusion and thickness with diabetes [60,61,62]. Ultimately, addressing whether this abnormal vascular response in dark adaptation contributes to DCP non-perfusion and photoreceptor abnormalities [63] in more advanced DR will be an interesting avenue for future clinical investigation. Further studies aimed at quantifying choroidal perfusion in diabetes are critically needed to complement our understanding of outer retinal responses in the dark.

While the compromised DCP response is the only abnormality we detected in the dark in mild NPDR individuals, during the transition to ambient light, neurovascular coupling in all three vascular plexuses appears to be affected in diabetic individuals. Our data in age-matched healthy control subjects showed similar general trends during ambient light transition as we previously reported: SCP large vessel dilation and MCP and DCP constriction, along with increased AFI across all three layers (Figure 5 and Figure 6) [35,36,37]. However, the DCP constriction in ambient light in our current healthy study participants was not as dramatic as our previous studies in young healthy subjects, who had more than double the DCP VD reduction compared to the current data [35,36,37]. This could potentially be explained by the previously reported age-related decrement in neurovascular coupling responses [33].

From the OCTA of healthy controls, the increase in SCP VD in light is attributable to large vessel dilation, as shown by the relatively stable VLD, which usually reflects changes in the capillaries [35]. In healthy individuals, the dilation of the larger superficial vessels, leading to increased flow to the inner retina, is presumably responsive to increased retinal ganglion cell activity and metabolic demand with illumination [35,38]. However, our diabetic groups showed trends of complete reversal of SCP and MCP VD responses to light with additional DCP reversal in the mild NPDR group that was significant during at least one timepoint. The significantly decreased SCP VD in both diabetic groups at 5 min compared to controls suggests a relative superficial non-dilation or constriction in ambient light, with both the large and small vessels affected in DM no DR individuals, who also demonstrated decreased SCP VLD at 50 s. The inappropriate superficial vascular constriction in our diabetic groups may play a potential role in recurrent episodes of ischemia of the inner retina in diabetics during the transition to ambient light. In addition, it is likely that this ischemia is further exacerbated by the significantly lower baseline SCP VD in our mild NPDR group.

Our finding of significantly decreased SCP VD at 5 min in the absence of differences in GCC thickness between diabetics and controls suggests that the lower superficial vascular densities in diabetics reflect an inappropriate response to light rather than an appropriately attenuated vascular response in the context of neuronal loss in diabetes. In line with previous findings by Lasta et al. [64] who showed neurovascular alterations before neural dysfunction, our results support the idea that neurovascular coupling deficits may occur before detectable structural neuronal deficits on OCT. We acknowledge that we were unable to evaluate ganglion cell function in this study, but we believe that that OCT structural analysis would have revealed significant thinning if functional changes were present at this point [65,66].

During the transition to ambient light, the DM no DR group also showed a trend of attenuation in AFI rise that did not reach statistical significance, while the mild NPDR participants demonstrated a qualitative trend of complete reversal of AFI responses across all three layers that was significantly decreased in the DCP at 5 min compared to controls (Figure 6). In the context of a significant SCP VD decrease in the diabetic groups during the transition to ambient light, the attenuated AFI responses may reflect an overall limited blood flow across all layers due to SCP large vessel constriction. The large superficial retinal arterioles are interconnected with the three vascular beds [67] and thus a constriction of the superficial arterioles could restrict flow across all vascular layers (Figure 6). The decreased DCP AFI at 5 min in the mild NPDR individuals in combination with decreased SCP VD at the same timepoint supports this explanation. Consequently, the significant MCP dilation in both diabetic groups and the DCP dilation in mild NPDR individuals could suggest a reactive vasodilation in the middle capillaries to counteract the overall decreased flow [68,69].

Our study expands upon previous evidence of compromised neurovascular coupling in early diabetes [21,22,33,70], with additional insights into the differential responses of the three macular plexuses to the relevant metabolic demand. Studies using retinal vessel analyzer and laser Doppler flowmetry have demonstrated a decreased arteriolar and venular vasodilation to flicker stimulus, as well as an inappropriately attenuated vasoconstriction to hyperoxia in early diabetes [21,22,29,33,71]. Mandecka et al. showed that diminished flicker-induced vasodilation was present in DM no DR individuals and increased with the severity of DR [33]. Furthermore, using a dynamic vessel analyzer, Lim et al. demonstrated that poor vasodilation to flicker stimulus was predictive of disease progression at one year follow up [21]. Beyond changes detectable at the arteriolar level, our current study highlights the utility of OCTA in revealing the distinct neurovascular dynamics in individual macular capillary plexuses of diabetic patients. Taken together, these studies reveal an important role for incorporating functional vascular imaging in the study of diseases like diabetes. Future investigations may consider incorporating AOSLO, which could provide an even higher resolution understanding of local neurovascular regulation. In healthy controls, Zhong et al. has demonstrated the effects of metabolic demand on local vascular changes by showing increased red blood cell velocity on AOSLO to flicker stimulus presented to a downstream region corresponding to the retinal artery of interest as compared to outside the region, providing a strong rationale for incorporating these approaches to the study of eyes with DR [72].

The strengths of our study include the distinctive design that allowed for the investigation of dark adaptation and light transition in all three vascular plexuses. The rigor of our study is reinforced by multiple quality control measures, including: diagnosing patients both clinically and on recent fundus photography; stringent image quality exclusion based on both SSI and Q-score, as well as review of motion and decentration artifacts; two separate graders of OCTA parameters; and, most importantly, the statistical adjustment for Q-score and age as well as for multiple comparisons. The biggest limitation of our study is the relatively small sample size and the complexity of the imaging protocol that led to the exclusion of data points that did not meet quality standards within experiments. In addition, the use of multiple comparison adjustments may decrease type I errors but increase type II errors and thus have potentially introduced false negatives. This study was also subject to the limitations of OCTA, such as minor projection artifacts and decentration of the scan on the FAZ that did not meet the threshold for exclusion. Lastly, our study is limited by a cross-sectional design that cannot provide temporal information regarding the predictive nature of our findings on disease progression, an important question to be addressed by future longitudinal study designs.

## 5. Conclusions

In summary, we show reversed retinal hemodynamic responses in the three macular vascular plexuses during dark adaptation and ambient light transition in individuals with DM no DR and mild NPDR. In the dark, mild NPDR individuals show significantly lower DCP VD relative to healthy individuals, which may theoretically increase photoreceptor susceptibility to ischemia in these eyes. During the transition to ambient light, we found a significant decrease of the SCP VD and increase of the MCP VD in both the DM no DR and mild NPDR groups, as well dilation of the DCP in those with mild NPDR. In combination with the general trends of AFI attenuation and reversal during light adaptation in the DM no DR and mild NPDR groups respectively, the VD data may suggest an inadequate inner retinal vascular supply and a hemodynamic mismatch to the metabolic demands during light adaptation. These findings illustrate the complexity and importance of neurovascular coupling defects in early DR and the intricacy revealed by investigating all three macular vascular plexuses. Additional studies are needed to explore how characterizing OCTA neurovascular findings may be translated into understanding neurovascular effects on retinal function and disease progression, which could ultimately play a role in disease screening and risk prediction.

## Figures and Tables

**Table 1:** Demographic Information.

Characteristic	Control (*n* = 19)	DM No DR (*n* = 14)	Mild NPDR (*n* = 11)	*p*-Value
Gender (F/M)	10/9	8/6	7/4	
Age (years) ^†^	44.5 ± 3.0	55.0 ± 3.1	50.4 ± 4.7	0.11
Diabetes type				
Type 1, *n* (%)		2 (14.3%)	5 (45.5%)	
Type 2, *n* (%)		12 (85.7%)	6 (54.5%)	
Disease duration (years) ^†^		8.4 ± 1.3	13.8 ± 3.6	0.44
Arterial hypertension, *n* (%)	2 (10.5%)	4 (28.6%)	3 (27.3%)	
MAP (mmHg) ^†^	83.5 ± 2.0	93.5 ± 2.8	91.3 ± 2.5	0.008 *
HbA1c (%) ^†^		6.6 ± 0.14	7.3 ± 0.29	0.024 *
Glucose (mg/dL) ^†^	95.68 ± 2.4	119.6 ± 6.8	144.8 ± 5.4	<0.01 *
Insulin dependent, *n* (%)		3 (21.4%)	5 (45.5%)	

* Statistical significance (*p*–value < 0.05); ^†^ values reported as mean ± standard error. Abbreviations: DM—diabetes mellitus, DR—diabetic retinopathy, NPDR—non-proliferative diabetic retinopathy, *n*—number of individuals, MAP—mean arterial blood pressure, mmHg—millimeters of mercury, F—female, M—male, mg/dL—milligram per deciliter, HbA1c—hemoglobin A1c.

## Supplementary Materials

The following are available online at https://www.mdpi.com/2077-0383/9/11/3523/s1, Supplementary Table S1: Parafoveal optical coherence tomography angiographic parameters at baseline and after dark adaptation, Supplementary Table S2: Parafoveal optical coherence tomography angiographic parameters during transition to ambient light.
