# Supplementary material for: Reversed Neurovascular Coupling on Optical Coherence Tomography Angiography Is the Earliest Detectable Abnormality before Clinical Diabetic Retinopathy
Source: J Clin Med. 2020 Oct 31;9(11):3523. doi: 10.3390/jcm9113523 (PMC7692675; doi:10.3390/jcm9113523)
Supplement: Supplementary file 1 [file jcm-09-03523-s001.pdf]

Supplementary Table S1. Parafoveal optical coherence tomography angiographic parameters at baseline and after dark adaptation.

. OCT or OCTA parameter	Baseline			Pairwise Comparisons P values		
	Control	DM no DR	mild NPDR	Control vs. DM no DR	Control vs. Mild NPDR	DM no DR vs. Mild NPDR
SCP AFI	0.423 ± 0.008	0.418 ± 0.009	0.425 ± 0.009	1	0.87	1
MCP AFI	0.432 ± 0.009	0.422 ± 0.010	0.428 ± 0.011	1	0.79	1
DCP AFI	0.42 ± 0.010	0.411 ± 0.012	0.413 ± 0.013	1	1	0.91
VLD, %	19.9 ± 0.50	21.7 ± 0.60	18.4 ± 0.70	0.051	**< 0.01**†	**0.003**†
SCP VD, %	47.63 ± 0.85	50.44 ± 0.96	44.37 ± 1.06	**0.036**†	**0.035**†	**< 0.001**†
MCP VD, %	46.00 ± 0.98	43.46 ± 1.11	42.22 ± 1.22	0.15	0.067	0.47
DCP VD, %	42.03 ± 1.06	42.56 ± 1.89	44.54 ± 2.08	0.94	1	0.75
GCC Thickness, μm	110.90 ± 2.02	110.31 ± 2.37	109.41 ± 2.63	0.85	1	1
**OCTA Parameter**	**Dark vs. Baseline***			**Pairwise Comparisons P values**		
	**Control**	**DM no DR**	**mild NPDR**	**Control vs. DM no DR**	**Control vs. Mild NPDR**	**DM no DR vs. Mild NPDR**
SCP AFI	-0.020- ± 0.008	-0.016 ± 0.009	-0.004 ± 0.010	0.73	0.2	0.36
MCP AFI	-0.026 ± 0.011	-0.015 ± 0.012	-0.003 ± 0.013	0.52	0.18	0.52
DCP AFI	-0.036 ± 0.013	-0.026 ± 0.014	-0.010 ± 0.015	0.51	0.14	0.42
VLD, %	0.70 ± 0.40	0.80 ± 0.50	0.0 ± 0.50	1	0.95	0.42
SCP VD, %	-0.22 ± 0.614	-0.056 ± 0.679	0.15 ± 0.72	0.87	1	1
MCP VD, %	1.06 ± 1.20	1.78 ± 1.33	-0.086 ± 1.40	0.7	0.81	1
DCP VD, %	4.18 ± 2.38	8.09 ± 2.63	-5.38 ± 2.77	0.29	**0.021**†	**< 0.01**†

*Dark measurements are analyzed and shown relative to baseline as dark minus baseline. †Statistical significance (P value < 0.05) Optical coherence tomography angiography parameters are reported as mean ± standard error. Abbreviations: DM—diabetes mellitus, DR—diabetic retinopathy, NPDR—non-proliferative diabetic retinopathy, SCP—superficial capillary plexus, MCP—middle capillary plexus, DCP—deep capillary plexus, AFI—adjusted flow index, VD—vessel density, VLD—vessel length density, GCC—ganglion cell complex.

Supplementary Table S2. Parafoveal optical coherence tomography angiographic parameters during transition to ambient light.

OCTA Parameter	50s Light vs. Dark*			Pairwise Comparisons P values		
	Control	DM no DR	mild NPDR	Control vs. DM no DR	Control vs. Mild NPDR	DM no DR vs. Mild NPDR
SCP AFI	0.001 ± 0.006	0.001 ± 0.007	-.020 ± 0.007	0.95	0.096	0.08
MCP AFI	0.003 ± 0.008	0.001 ± 0.010	-0.024 ± 0.010	0.889	0.123	0.117
DCP AFI	0.002 ± 0.008	-0.003 ± 0.010	-0.028 ± 0.010	0.684	0.072	0.123
VLD, %	0.70 ± 0.20	-0.30 ± 0.30	0.20 ± 0.30	**0.042**[†]	0.255	0.297
SCP VD, %	1.08 ± 0.50	-0.51 ± 0.61	-0.81 ± 0.63	0.084	0.075	0.733
MCP VD, %	-3.33 ± 0.99	1.56 ± 1.22	1.48 ± 1.25	**0.012**[†]	**< 0.01**[†]	0.962
DCP VD, %	-2.96 ± 1.94	-5.45 ± 2.38	6.64 ± 2.44	0.43	**< 0.01**[†]	**0.003**[†]
	**2 mins Light vs. Dark***			**Pairwise Comparisons P values**		
**OCTA Parameter**	**Control**	**DM no DR**	**mild NPDR**	**Control vs. DM no DR**	**Control vs. Mild NPDR**	**DM no DR vs. Mild NPDR**
SCP AFI	0.009 ± 0.007	0.001 ± 0.008	-.008 ± 0.009	0.71	0.50	0.51
MCP AFI	0.011 ± 0.009	0.00 ± 0.011	-0.010 ± 0.013	0.70	0.59	0.58
DCP AFI	0.011 ± 0.010	-0.001 ± 0.012	-0.015 ± 0.014	0.64	0.37	0.46
VLD, %	0.10 ± 0.30	-0.60 ± 0.30	0.80 ± 0.40	0.14	0.17	**0.024**[†]
SCP VD, %	0.70 ± 0.60	-1.71 ± 0.73	-0.05 ± 0.83	0.24	0.53	0.50
MCP VD, %	-2.33 ± 1.08	1.61 ± 1.30	-0.64 ± 1.49	0.09	0.36	0.40
DCP VD, %	-1.68 ± 2.38	-8.86 ± 2.87	1.68 ± 3.0	0.11	0.41	0.07
	**5 mins Light vs. Dark***			**Pairwise Comparisons P values**		
**OCTA Parameter**	**Control**	**DM no DR**	**mild NPDR**	**Control vs. DM no DR**	**Control vs. Mild NPDR**	**DM no DR vs. Mild NPDR**
SCP AFI	0.025 ± 0.008	0.011 ± 0.010	-.008 ± 0.011	0.28	0.06	0.29
MCP AFI	0.029 ± 0.010	0.006 ± 0.012	-0.009 ± 0.013	0.28	0.099	0.39
DCP AFI	0.037 ± 0.011	0.008 ± 0.013	-0.014 ± 0.015	0.2	0.036	0.27
VLD, %	-0.20 ± 0.30	-1.10 ± 0.30	0.60 ± 0.40	0.065	0.12	**0.003**[†]
SCP VD, %	1.71 ± 0.53	-0.85 ± 0.62	-0.84 ± 0.68	**0.015**[†]	**0.009**[†]	0.99
MCP VD, %	-1.63 ± 1.10	0.28 ± 1.28	-0.38 ± 1.42	0.86	0.74	0.73
DCP VD, %	-6.74± 2.22	-3.86 ± 2.60	5.57 ± 2.86	0.42	**< 0.01**[†]	0.029
	**15 mins Light vs. Dark***			**Pairwise Comparisons P values**		

OCTA Parameter	Control	DM no DR	mild NPDR	Control vs. DM no DR	Control vs. Mild NPDR	DM no DR vs. Mild NPDR
SCP AFI	0.018 ± 0.007	0.06 ± 0.008	-.002 ± 0.009	0.46	0.29	0.52
MCP AFI	0.015 ± 0.009	0.05 ± 0.010	-0.001 ± 0.011	0.71	0.76	0.68
DCP AFI	0.021 ± 0.010	0.014 ± 0.012	0.001 ± 0.013	0.64	0.68	0.71
VLD, %	0.20 ± 0.70	0.40 ± 0.80	0.60 ± 0.90	1	2.2	0.87
SCP VD, %	1.50 ± 0.57	-0.79 ± 0.66	-0.20 ± 0.73	**0.045**[†]	0.11	0.55
MCP VD, %	-3.48 ± 1.30	-0.87 ± 1.52	-0.10 ± 1.66	0.32	0.36	0.74
DCP VD, %	-3.59 ± 2.72	-11.50 ± 3.20	3.98 ± 3.50	0.11	0.096	**< 0.01**[†]

*OCTA measurements in ambient light time points are analyzed and shown relative to dark as light minus dark. [†]Statistical significance (P value < 0.05). Optical coherence tomography angiography parameters are reported as mean ± standard error. Abbreviations: DM—diabetes mellitus, DR—diabetic retinopathy, NPDR—non-proliferative diabetic retinopathy, SCP—superficial capillary plexus, MCP—middle capillary plexus, DCP—deep capillary plexus, AFI—adjusted flow index, VD—vessel density, VLD—vessel length density.
